# Control of glycemia and blood pressure in British adults with diabetes mellitus and subsequent therapy choices: a comparison across health states

**DOI:** 10.1186/s12933-018-0673-4

**Published:** 2018-02-12

**Authors:** Finlay A. McAlister, Brendan Cord Lethebe, Caitlin Lambe, Tyler Williamson, Mark Lowerison

**Affiliations:** 1 Division of General Internal Medicine, 5-134C Clinical Sciences Building, University of Alberta, 11350 83 Avenue, Edmonton, AB T6G 2G3 Canada; 2 Patient Health Outcomes Research and Clinical Effectiveness Unit, 5-134C Clinical Sciences Building, University of Alberta, 11350 83 Avenue, Edmonton, AB T6G 2G3 Canada; 3 Clinical Research Unit, Cumming School of Medicine, University of Calgary, Calgary, Canada

**Keywords:** Glycemic control, Hypertension, Database research, Pharmaco-epidemiology, Diabetes mellitus, Treatment, Targets, Deintensification

## Abstract

**Background:**

To examine the intensity of glycemic and blood pressure control in British adults with diabetes mellitus and whether control levels or treatment deintensification rates differ across health states.

**Methods:**

Retrospective cohort study using primary care electronic medical records (the United Kingdom Health Improvement Network Database) for adults with diabetes diagnosed at least 6 months before the index HbA1C and systolic blood pressure (SBP) measurements (to give their primary care physicians time to achieve treatment goals). We used prescribing records for 6 months pre/post the index measurements to determine who had therapy subsequently deintensified (based on “glycemic therapy score” and “antihypertensive therapy score” derived from number and dosage of medications).

**Results:**

Of 292,170 individuals with diabetes, HbA1C < 6% or SBP < 120 mmHg after at least 6 months of management was less common in otherwise fit patients (15.0 and 12.7%) than in those who were mildly frail (16.6 and 13.2%) or moderately–severely frail (20.2 and 17.0%, both p < 0.0001). In the next 6 months, only 44.7% of those with HbA1C < 6% had glycemic therapy reduced (44.4% of fit, 47.1% of mildly frail, and 41.5% of moderate-severely frail patients) and 39.8% of those with SBP < 120 had their antihypertensives decreased (39.3% of fit, 43.0% of mildly frail, and 46.7% of moderate-severely frail patients). On the other hand, more individuals exhibited higher than recommended levels for HbA1C or SBP after the first 6 months of therapy (37.3, 33.4, and 31.3% of fit, mildly frail, and moderately–severely frail patients had HbA1C > 7.5% and 46.6, 51.4, and 48.5% had SBP > 140 mmHg). The proportions of patients with HbA1C or SBP out of recommended treatment ranges changed little 6 months later despite frequent (median 14 per year) primary care visits.

**Conclusions:**

Glycemic and hypertensive control exhibited statistically significant but small magnitude differences across frailty states. Medication deintensification was uncommon, even in frail patients below SBP and HbA1C targets. SBP levels were more likely to be outside recommended treatment ranges than glycemic levels.

*Trial registration* As this study is a retrospective secondary analysis of electronic medical record data and not a health care intervention trial it was not registered

**Electronic supplementary material:**

The online version of this article (10.1186/s12933-018-0673-4) contains supplementary material, which is available to authorized users.

## Background

Choosing Wisely (https://www.choosingwisely.org), the “Do not do” recommendations from NICE (https://www.nice.org.uk), and various guideline bodies have raised the issue of whether some individuals with type 2 diabetes mellitus may be over-treated in light of observational studies suggesting that the relationship between HbA1C levels and poor outcomes is U-shaped [1, 2]. Certainly, beyond slowing of diabetic retinopathy progression, the benefits of intensive glucose control are uncertain for patients with type 2 diabetes, and are offset by increased risks of hypoglycemic events [and even all-cause mortality in the Action to Control Cardiovascular Risk in Diabetes (ACCORD) trial], especially in the elderly or those with substantial comorbidities [3–5]. Although diabetes guidelines recommend individualization of glycemic control with HbA1c goals of less than 7.0% in healthy individuals (and < 7.5% in healthy patients older than 65 years) with less stringent targets in patients with comorbidities or frailty, several recent analyses from the United States demonstrated that many adults with type 2 diabetes had HbA1C ≤ 7% with little difference in glycemic control across health states [6–8]. Moreover, only a minority of patients who were potentially over-treated subsequently had their glycemic therapy deintensified [9–11].

In a similar vein, current hypertension guidelines [12, 13] advise individualization of antihypertensive therapy and caution against overtreatment in patients with type 2 diabetes since the ACCORD trial did not demonstrate any reduction in major cardiovascular events with intensive control of systolic blood pressure (SBP) but instead an increase in serious adverse events [14]. A recent systematic review of 49 antihypertensive trials in diabetes also demonstrated that benefit from treatment was only present in those patients with SBP > 140 mmHg [15]. Despite this trial evidence and studies suggesting risk with lower SBP, especially in older patients with comorbidities [16–18], a recent report from the US Veterans Health Administration demonstrated that half of patients with type 2 diabetes had SBP less than 130 mmHg and over one-third had SBP less than 120 mmHg [9].

The purpose of this study is to examine risk factor control (glucose and SBP) in a large cohort of patients with diabetes cared for by UK primary care physicians, to examine whether control varied by health status, and to explore whether patterns of treatment deintensification varied by risk factor level and/or health status.

## Methods

### Cohort selection

We identified all patients aged 20 years or older with diabetes mellitus in the health improvement network (THIN) database based on read clinical encounter codes (akin to diagnostic codes in North American physician billing data) or any diabetic prescriptions (using free word searching in the ontology navigator to select drugs identified by FM as being glucose lowering drugs in the British National Formulary)—full list available from author BCL on request. THIN data collection began in 2003 and by September 2015 more than 670 NHS primary care practices had contributed data from over 14 million patients to THIN, with 4.4 million patients (approximately 7% of the UK population) actively registered with and followed over time by THIN practices. The THIN dataset is constructed from deidentified data collected from each participating primary care physician’s electronic medical record system using standardised coding systems and only reflects those events deemed relevant to the patient’s care by their physician since data are recorded for practice and patient management and not directly for research purposes. Specialty clinics are not included in the THIN dataset we accessed but if specialists made recommendations then those accepted by the attending primary care physician were captured. This dataset has been used in over 600 published studies thus far, is representative of the UK population, and the accuracy of diagnostic coding for chronic conditions, such as diabetes, is high, particularly if one combines physician-assigned READ codes with prescription data [19, 20].

Our cohort represents a mix of prevalent and incident cases of diabetes seen at 633 THIN-participating clinics between 2003 and 2015. In order to be eligible for this study, patients in the cohort had to have a HbA1C measurement and a SBP measurement at least 6 months after the initial diagnosis of diabetes (to give their physicians time to initiate and titrate management)—for the purposes of this study, these values are defined as the index measurements.

### Definition of health status

For each patient, we calculated the electronic frailty index (eFI) score using all visits and prescriptions in the 24 months prior to and including the date that the HbA1C used to define their glycemic control was drawn (see below). The eFI is based on the cumulative deficit frailty model and can be used to group patients into categories of fit (eFI score ≤ 0.12), mild (eFI 0.13–0.24), moderate (eFI 0.25–0.36), and severe (eFI > 0.36) frailty [21]. The eFI was developed in the ResearchOne Database and externally validated in the THIN dataset for patients older than 65 and demonstrates good discrimination for risk of mortality (c statistics of 0.72 and 0.74 in different cohorts), hospitalization (c statistics 0.66 and 0.71 in different cohorts) and nursing home admission (c statistic 0.74 in the ResearchOne cohort). As there were very few patients with eFI scores in the severe range in our cohort (n = 12), we grouped moderate and severe frailty for this analysis.

### Definition of glycemic control

We used the HbA1C for each patient recorded at least 6 months after the initial diagnosis of diabetes to define the index date for the glycemia analyses. Mirroring prior publications [2, 14, 15], we grouped treated HbA1C into < 6, 6.0–6.4, 6.5–6.9, 7.0–7.5, and > 7.5%. We also examined the proportion of patients who met a published definition within the US Veterans Affairs Diabetes Quality Enhancement Research Initiative (https://www.queri.research.va.gov) of “definite glycemic overtreatment”: HbA1C < 7%, receiving a sulfonylurea or insulin, and (i) 75 years or older, or (ii) eGFR < 60 mL/min, or (iii) dementia [8]. In a sensitivity analysis we examined glycemic control in patients who were actively taking glucose lowering medications at the time of the index HbA1C measurement.

### Definition of blood pressure control

We used the SBP closest to the index HbA1C date to define blood pressure control. Based on prior publications [9], we categorized SBP as < 120, 120–129, 130–139, and ≥ 140 mmHg. We also examined the proportion of patients who met a proposed definition of “hypertension overtreatment” for patients with diabetes (also from the VA Diabetes Quality Enhancement Research Initiative, https://www.queri.research.va.gov): SBP < 130 mmHg and (i) receiving at least three antihypertensive drugs, or (ii) starting an additional antihypertensive drug within 90 days of the index SBP measurement, or (iii) increasing dose of antihypertensive drugs within 90 days of the index SBP measurement [22]. In a sensitivity analysis we examined SBP control in patients who were actively taking antihypertensive medications at the time of the index SBP measurement.

### Definition of deintensification

We used the dosgval field in the THIN database (which lists the 25,000 most common drug doses and daily frequencies) to calculate a “glycemic therapy score” and “antihypertensive therapy score” for each patient using prescription records for the 6 months before and after the index measurements. We examined the frequency of treatment deintensification defined as a lower glycemic therapy score (analysis 1) or antihypertensive therapy score (analysis 2) after the index HbA1C or SBP compared to prior to the index measurements. Note that a lower score was obtained if patients stopped (no refill) or were prescribed a lower dose of glucose lowering drugs (analysis 1) or antihypertensive agents (analysis 2) in the 6 months after the index HbA1C (analysis 1) or SBP measurement (analysis 2). As prescription records cannot define daily dose of subcutaneous insulin that the patient actually takes, in a sensitivity analysis we excluded any patients using insulin from the glycemic therapy analysis—as results were not different from the main analysis we did not report them separately. For analysis 1 (glycemic therapy), we compared deintensification rates for patients with index HbA1C < 6.0, 6.0–6.4, 6.5–6.9, 7.0–7.5, and > 7.5%. For analysis 2 (antihypertensive therapy), we compared deintensification rates for patients with index SBP < 120, 120–129, 130–139, and ≥ 140 mmHg.

### Covariates

The specific variables included are outlined in Table 1—we identified comorbidities using previously validated read codes assigned by each patient’s primary care physician on their EMR [20]. In patients who did not have some of the laboratory data measured, the missing indicator approach was utilized (patients with missing HbA1C or SBP were excluded).

**Table 1:** Patient socio-demographics, healthcare utilization, lab results and prescribed drugs up until the time of the index HbA1C measurement (6 months after diagnosis), stratified according to patient health status

Characteristics	Overall (n = 292,170)	Otherwise fit (n = 270,068)	Mild frailty (n = 21,448)	Moderate or severe frailty (n = 654)	p value
Age, mean (SD)	61.7 (15.6)	60.8 (15.5)	71.7 (12.2)	77.2 (11.2)	< 0.0001
Female, % (n)	45.2 (132,167)	44.3 (119,595)	56.7 (12,169)	61.6 (403)	< 0.0001
Number of primary care physician visits in the year prior to index measurement, median (Q1, Q3)	14 (8, 21)	13 (7, 20)	25 (16, 36)	39 (29, 54)	< 0.0001
Charlson score, mean (SD)	1.10 (0.92)	1.04 (0.86)	1.85 (1.28)	2.73 (1.6)	< 0.0001
At least two chronic comorbidities (from the list below)	8.0 (23,464)	5.1 (13,768)	42.8 (9187)	77.8 (509)	< 0.0001
Specific comorbidities (not mutually exclusive)					
Hypertension	15.6 (45,470)	14 (37,695)	35 (7516)	39.6 (259)	< 0.0001
Chronic kidney disease	1.1 (3166)	0.8 (2054)	4.8 (1038)	11.3 (74)	< 0.0001
Ischemic heart disease (including prior myocardial infarction or CABG)	6.0 (17,501)	4.4 (11,888)	24.9 (5334)	42.7 (279)	< 0.0001
Heart failure	1.8 (5256)	0.8 (2246)	12.8 (2748)	40.1 (262)	< 0.0001
Cerebrovascular disease	2.1 (6133)	1.7 (4701)	6.3 (1360)	11.0 (72)	< 0.0001
COPD	3.3 (9523)	2.5 (6875)	11.6 (2489)	24.3 (159)	< 0.0001
Cancer	0.1 (347)	0.1 (270)	0.3 (75)	0.3 (2)	< 0.0001
Depression	4.6 (13,484)	4.3 (11,585)	8.4 (1795)	15.9 (104)	< 0.0001
Dementia	0.6 (1818)	0.4 (1118)	3.0 (647)	8.1 (53)	< 0.0001
Urinary incontinence	1.5 (4369)	0.9 (2528)	8.0 (1707)	20.5 (134)	< 0.0001
Arthritis	5.6 (16,476)	4.3 (11,687)	21.2 (4552)	36.2 (237)	< 0.0001
Diabetes complications					
Retinopathy	5.2 (15,131)	4.9 (13,114)	9.1 (1951)	10.1 (66)	< 0.0001
Neuropathy	0.4 (1031)	0.3 (823)	0.9 (201)	1.1 (7)	< 0.0001
Nephropathy	0.2 (566)	0.1 (365)	0.9 (195)	0.9 (6)	< 0.0001
Any of the above	5.6 (16,396)	5.2 (14,097)	10.4 (2224)	11.5 (75)	< 0.0001
Physical measurements closest to index HbA1C date					
SBP	138.2 (19.4)	138.1 (19.3)	139.9 (21.3)	137.9 (22.6)	< 0.0001
DBP	79.2 (11.0)	79.4 (10.9)	77.4 (11.8)	74.5 (12.5)	< 0.0001
BMI	30.0 (6.9)	30.17 (6.9)	28.8 (7.1)	26.0 (6.8)	< 0.0001
Lab values					
Estimated glomerular filtration rate category (mL/min)	70.7 (18.1)	71.3 (17.8)	62.7 (19.5)	56.5 (20.7)	< 0.0001
< 30	1.6 (833)	1.3 (639)	4.8 (181)	9.8 (13)	< 0.0001
30 to < 60	17.6 (9089)	16.3 (7752)	33.7 (1275)	47 (62)	< 0.0001
≥ 60	80.7 (41,581)	82.4 (39,192)	61.6 (2332)	43.2 (57)	< 0.0001
Mean (SD) total cholesterol (mmol/L)	4.77 (3.62)	4.78 (3.73)	4.66 (1.69)	4.41 (1.17)	0.007
Mean (SD) triglycerides (mmol/L)	2.02 (1.97)	2.02 (2)	1.96 (1.41)	1.93 (1.41)	0.05
Mean (SD) LDL cholesterol (mmol/L)	2.6 (2.92)	2.61 (3.02)	2.43 (0.95)	2.15 (0.75)	0.001
Mean (SD) HbA1c (%)	7.37 (1.64)	7.38 (1.64)	7.23 (1.56)	7.10 (1.46)	< 0.0001
Drugs prescribed within 120 days preceding the index HbA1C measurement (not mutually exclusive)					
Insulin	17.0 (49,623)	16.9 (45,740)	17.5 (3764)	18.2 (119)	0.05
Sulfonylurea	24.2 (70,666)	23.6 (63,625)	31.7 (6789)	38.5 (252)	< 0.0001
Metformin	48.2 (140,964)	48.4 (130,620)	46.9 (10,058)	43.7 (286)	< 0.0001
Thiazolidinedione	4.2 (12,376)	4.2 (11,441)	4.2 (911)	3.7 (24)	0.77
Dipeptidyl peptidase-4 inhibitors	1.0 (2899)	1.0 (2684)	1.0 (207)	1.2 (8)	0.77
Other antidiabetic agents	1.4 (4088)	1.4 (3756)	1.5 (318)	2.1 (14)	0.15
ACE inhibitor/ARB	46.8 (136,812)	45.4 (122,711)	63.6 (13,637)	70.9 (464)	< 0.0001
Statin	51.9 (151,782)	51.3 (138,509)	60.1 (12,882)	59.8 (391)	< 0.0001
Beta blocker	18.1 (52,776)	17.7 (47,821)	22.5 (4818)	20.9 (137)	< 0.0001
Other antihypertensive agents	71.4 (208,666)	70.5 (190,427)	82.5 (17,696)	83.0 (543)	< 0.0001
Antiplatelet agent	37.9 (110,589)	36 (97,216)	60.2 (12,908)	71.1 (465)	< 0.0001

Patient characteristics are reported as means and standard deviations for continuous variables and proportions for categorical variables (with numbers in brackets)

Health status defined by eFI score: fit (≤ 0.12), mild frailty (eFI 0.13–0.24), moderate frailty (eFI 0.25–0.36), and severe frailty (eFI > 0.36) [21].

### Statistical analysis

Patient characteristics were reported as means and standard deviations for continuous variables and proportions for categorical variables. T tests and one-way ANOVA were used to compare quantitative values between groups. Chi squared tests were used to compare categorical and ordinal values between groups. 95% confidence intervals were calculated using the Student’s t-distribution for quantitative values and the normal approximation to the binomial distribution was used for proportions. As we conducted multiple comparisons the p value for statistical significance should be < 0.001 rather than < 0.05.

### Ethics

As we were using de-identified data, waiver of informed consent was granted by the University of Calgary Health Research Ethics Board (REB15-0203_REN3). This study was based on data from the THIN database obtained by the Cumming School of Medicine at the University of Calgary under license from IQVIA (IMS Quintiles VIA—see https://www.iqvia.com).

## Results

**Fig. 1:**
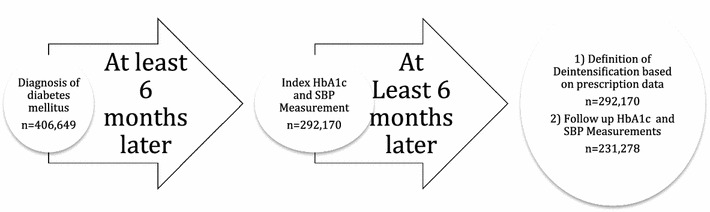
Study outlined and derivation of study sample

Of 406,649 individuals with diabetes, 297,589 had a HbA1C drawn and 292,703 had a SBP measured at least 6 months after their diabetes diagnosis; the 292,170 with both HbA1C and SBP in the Thin dataset after at least 6 months of diabetes formed the sample for this study (Fig. 1). Mean age was 61.7 years, median time to the index measurements was 354 days (IQR 254–701) after diabetes diagnosis, and the median number of primary care physician visits in the year prior to the index measurements was 14—ranging from 13 in otherwise fit individuals with diabetes up to 39 in those with moderate or severe frailty (Table 1). Of note, the median time between the index HbA1C and index SBP measurements was 12 days.

Most patients in the study sample were otherwise fit (270,068, 92.4%), 21,448 (7.3%) met the definition for mild frailty, 642 (0.2%) moderate frailty, and 12 were severely frail (we lumped moderate and severe frailty together in subsequent analyses). Comorbidities and health resource use were substantially higher as degree of frailty increased (Table 1). Diabetes complications (most commonly retinopathy) were not uncommon, even in the relatively healthy subgroups: 5.2% of otherwise fit patients and 10.3% of those classified as having mild frailty had a diabetes complication noted by the time of the index measurements.

### Risk factor control

The mean index HbA1C was 7.4% in otherwise fit patients, 7.2% in mildly frail patients, and 7.1% in moderately–severely frail patients (Table 1). While 37.0% of patients had index HbA1C > 7.5, 15.2% had HbA1C < 6% and 16.4% had HbA1C 6.0–6.4% with little change 6 months later: 37.7, 13.1, and 15.9% respectively (Table 2). Glycemic and SBP control were significantly different across health status strata (Table 2, both p < 0.001). The proportion of patients with HbA1C < 6.5% was lower in otherwise fit patients (31.3%) than in mildly frail patients (34.1%) or those who were moderately–severely frail (37.2%, p < 0.001). Although the sensitivity analysis limited to the 160,322 patients actively taking glucose lowering medications at the time of the index HbA1C measurement found slightly smaller proportions, the patterns were the same (26.3% vs. 29.4% vs. 32.7%, p < 0.0001, Additional file 1: Appendix Table S1). The VA definition of “definite glycemic overtreatment” [8] was met by 3.5% of patients.

**Table 2:** Glycemic and blood pressure levels in the first year after diagnosis of diabetes

	Overall (n = 292,170)	Otherwise fit (n = 270,068)	Mild frailty (n = 21,448)	Moderate or severe frailty (n = 654)	p value
*Index HbA1C (after at least 6 months of management)*					
< 6%	15.2 (44,293)	15.0 (40,602)	16.6 (3559)	20.2 (132)	< 0.0001
6.0–6.4%	16.4 (47,985)	16.3 (44,113)	17.5 (3761)	17 (111)	< 0.0001
6.5–6.9%	18.1 (53,020)	18.1 (48,844)	18.9 (4055)	18.5 (121)	0.01
7.0–7.5%	13.3 (38,765)	13.2 (35,777)	13.5 (2903)	13.0 (85)	0.48
> 7.5%	37.0 (108,107)	37.3 (100,732)	33.4 (7170)	31.3 (205)	< 0.0001

HbA1C at least 6 months after index HbA1C	Overall (n = 231,278)	Otherwise fit (n = 214,456)	Mild frailty (n = 16,398)	Moderate or severe frailty (n = 424)	p value
< 6%	13.1 (30,260)	12.9 (27,740)	14.9 (2450)	16.5 (70)	< 0.0001
6.0–6.4%	15.9 (36,735)	15.8 (33,848)	17.1 (2812)	17.7 (75)	< 0.0001
6.5–6.9%	18.9 (43,682)	18.8 (40,397)	19.6 (3211)	17.5 (74)	0.05
7.0–7.5%	14.4 (33,338)	14.5 (31,015)	13.8 (2269)	12.7 (54)	0.06
> 7.5%	37.7 (87,263)	38 (81,456)	34.5 (5656)	35.6 (151)	< 0.0001
*Index systolic blood pressure (after at least 6 months of management)*					
< 120 mmHg	12.7 (37,216)	12.7 (34,266)	13.2 (2839)	17.0 (111)	0.0003
120–129 mmHg	17.1 (49,825)	17.2 (46,582)	14.6 (3139)	15.9 (104)	< 0.0001
130–139 mmHg	23.3 (67,987)	23.5 (63,410)	20.8 (4455)	18.7 (122)	< 0.0001
140 mmHg or greater	46.9 (137,142)	46.6 (125,810)	51.4 (11,015)	48.5 (317)	< 0.0001

Systolic blood pressure 6 months after index	Overall (n = 247,199)	Otherwise fit (n = 228,210)	Mild frailty (n = 18,447)	Moderate or severe frailty (n = 542)	p value
< 120 mmHg	12.0 (29,578)	11.9 (27,091)	13.0 (2396)	16.8 (91)	< 0.0001
120–129 mmHg	17.3 (42,697)	17.4 (39,661)	15.9 (2940)	17.7 (96)	< 0.0001
130–139 mmHg	24.1 (59,621)	24.3 (55,557)	21.4 (3949)	21.2 (115)	< 0.0001
140 mmHg or greater	46.6 (115,303)	46.4 (105,901)	49.7 (9162)	44.3 (240)	< 0.0001

The mean SBP at the time of the index HbA1C measurement was 138.1 mmHg in otherwise fit patients, 139.9 mmHg in mildly frail patients, and 137.9 mmHg in moderately–severely frail patients (Table 1). While 46.9% of patients had index SBP ≥ 140 mmHg, 12.7% had SBP < 120 mmHg, and 17.1% had SBP 120–129 mmHg with very little change 6 months later: 46.6, 12.0, and 17.3% respectively. The proportion with SBP < 120 mmHg was lower in otherwise fit patients (12.7%) than in mildly frail patients (13.2%) or those who were moderately–severely frail (17.0%, p < 0.001). Although the sensitivity analysis limited to the 160,322 patients actively taking antihypertensive drugs at the time of the index SBP measurement found slightly smaller proportions, the patterns were the same (10.8% vs. 13.2% vs. 15.2%, p < 0.0001, Additional file 1: Appendix Table S1). The VA definition of “potential hypertension overtreatment” [22] was met by 11.8% of our cohort.

### Treatment deintensification

**Fig. 2:**
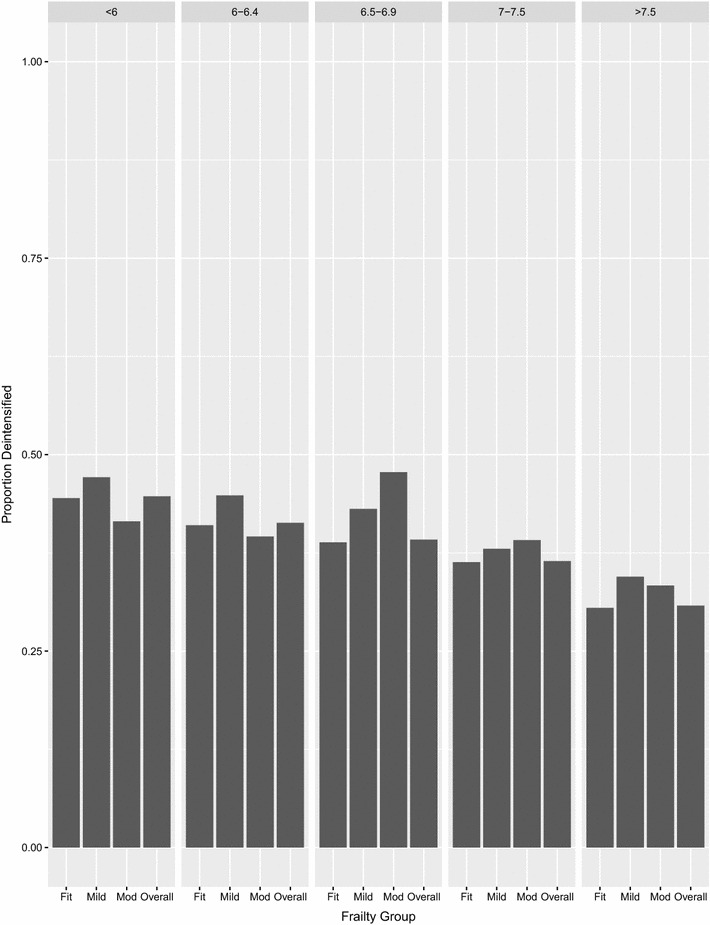
Proportion of patients with deintensification of glycemic treatment, by health status and within HbA1C strata

Of 154,691 patients being actively treated with hypoglycemic medications and with prescription data for 6 months before and 6 months after the index HbA1C, 56,129 (36.3%) had their glycemic therapy deintensified (Fig. 2). Followup HbA1C measurements 6 months later went up by a mean of 0.17 (95% CI 0.16–0.18) in patients who had therapy deintensified but went down by 0.06 (95% CI 0.05–0.07) in those who did not have deintensification of therapy. Deintensification was more common in patients with moderate-severe frailty (39.4%) or mild frailty (39.9%) than those who were otherwise fit (36.0%)—both p < 0.001. As expected, deintensification rates differed across HbA1C levels (44.7% in those with HbA1C < 6, 41.3% in those with HbA1C 6.0–6.4, 39.2% with HbA1C 6.5–6.9, 36.4% with HbA1c 7.0–7.5, and 30.8% of those with HbA1C > 7.5%, p < 0.001). There was no significant interaction between health state and HbA1C strata in the proportion of patients who had their therapy deintensified (p = 0.42 for the interaction term).

**Fig. 3:**
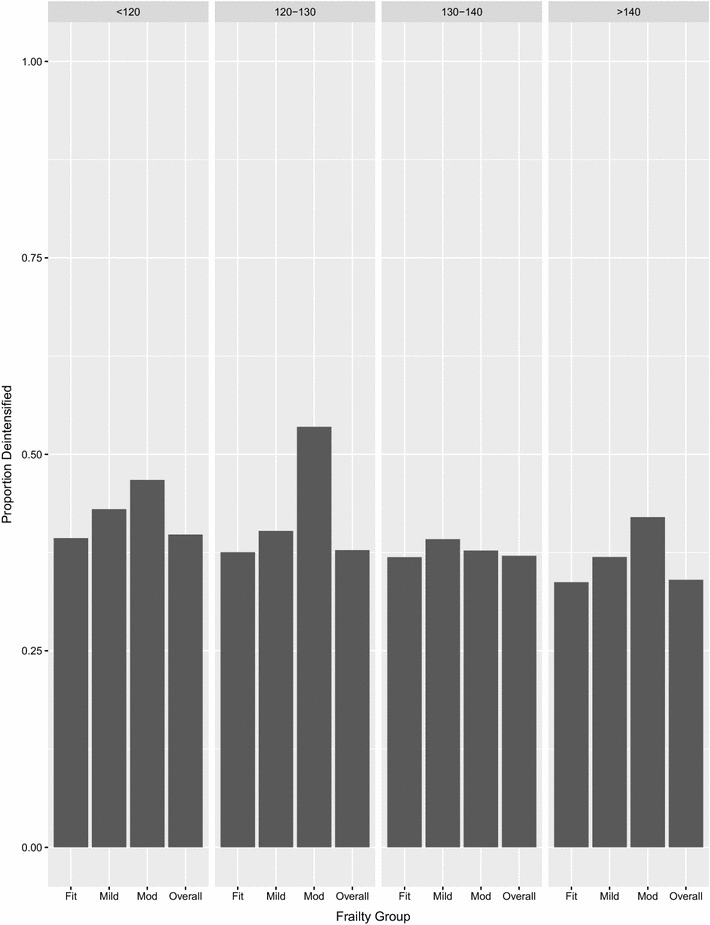
Proportion of patients with deintensification of antihypertensive treatment, by health status and within SBP strata

Of 187,852 patients being actively treated with antihypertensive medications and with prescription data for 6 months before and 6 months after the index SBP measurement, 67,625 (36.0%) of patients had their antihypertensive therapy deintensified with a statistically significant but small magnitude difference across BP levels (39.8% of those with SBP < 120, 37.8% of those with SBP 120–129, 37.1% of those with SBP 130–139, and and 34.0% of those with SBP > 140 mmHg)—Fig. 3. Deintensification was more common in patients with mild (38.8%) or moderate-severe (43.9%) frailty than those who were otherwise fit (35.7%)—both p < 0.001. There was no interaction between health state and SBP strata in the proportion of patients who had their therapy deintensified (p = 0.53).

Of note, 17,136 patients had both their glucose lowering and their antihypertensive therapies deintensified in the 6 months after the index measurements (approximately one-sixth of the 106,663 patients who had either therapy deintensified).

## Discussion

We found that glycemic and SBP control was similar for otherwise fit adults with diabetes as for frail patients, and that while nearly one-third of all diabetic individuals in this cohort of primary care-managed NHS patients had treated HbA1C < 6.5% or SBP < 130 mmHg, nearly half had HbA1C > 7.5% or SBP > 140 mmHg. This is in contrast to US Medicare data suggesting that overtreatment is more common than undertreatment in elderly Americans [23]. We also found that, although deintensification rates in this UK cohort are nearly double the 14–19% reported in US-based studies [8–10, 23], therapy was reduced in less than half of patients with low HbA1C or SBP levels, even if they were frail, despite frequent primary care follow-up (median 14 visits per year). On the other hand, it should be noted that even in otherwise fit individuals with diabetes, the proportions with HbA1C or SBP above recommended targets also differed little when re-measured 6 months later. This echoes an earlier study from the UK Clinical Practice Research Datalink which found that for the vast majority of patients with a new diagnosis of diabetes the HbA1C levels changed little after the first 6 months (followup was out to 24 months) and only 40% of patients with HbA1C levels above target had any therapy intensification in the subsequent year [24]. A recent analysis of 833 elderly individuals with type 2 diabetes mellitus in Canadian primary care practices reported findings similar to ours: approximately half of healthy older patients had HbA1C and SBP levels above target range, nearly half of frail patients exhibited potential overtreatment, and medication adjustments in either situation were uncommon [25]. Recent reports from China and a registry from 410 sites in 32 other countries also documented that less than half of patients with type 2 diabetes have their HbA1C and SBP controlled within recommended target ranges, although they did not distinguish between potential over- vs. under-treatment [26, 27].

Thus, our data highlight the importance of personalizing targets and multiple potential targets for quality improvement in primary care management of type 2 diabetes. For example, although the emphasis in diabetes professional education programs and guidelines is usually on glucose lowering options [4, 28], far more patients in our cohort exhibited uncontrolled hypertension than poor glycemic control—consistent with earlier studies in Canada, the United States, and the Netherlands [22, 28–31]. This pattern is concerning since blood pressure is the strongest driver of cardiovascular outcomes in diabetic individuals (with an attributable risk nearly three fold greater than glycemic levels in the Framingham study) [32]. The benefits of blood pressure lowering (at least to < 140 mmHg) [15, 17] exceed those of glucose lowering [3–5], and antihypertensive therapy is the most cost-effective of the cardiovascular prevention therapies in type 2 diabetes [33]. Moreover, it should be acknowledged that in all health states uncontrolled hypertension was far more common than over-controlled blood pressure. Thus, our study highlights the need for increased attention to the role of blood pressure control in improving outcomes for individuals with diabetes.

On the other side of the quality equation, although clinical performance measures have until recently almost exclusively focused on under-treatment and failure to meet targets [28, 34], there is increasing recognition of the potential harms that can arise from over-treatment. Intensive lowering of glucose and blood pressure have both been shown to be potentially harmful in individuals with diabetes [1–3, 11, 15]. Although we did not have access to complete data on emergency room visits or hospitalizations for our cohort, other studies have demonstrated that hypoglycemia is one of the most common presenting diagnoses in emergency departments for elderly individuals and results in more frequent and more severe hospitalizations than does hyperglycemia [35, 36]. Moreover, achieving intensive glycemic or SBP control requires polypharmacy which puts patients at increased risk for drug–drug interactions, poorer medication adherence, and reduced quality of life [16]. While efforts through Choosing Wisely, NICE, and the Veterans Affairs Hypoglycemia Safety Initiative to improve physician awareness of this issue are a welcome addition to the quality improvement landscape in diabetes, educational interventions alone are inadequate to overcome clinical inertia [37]. In fact, preliminary evidence suggests that clinical inertia is an even greater barrier against deintensifying therapy than introducing novel therapies [38, 39].

Although our study has several strengths due to the availability of detailed clinical data in a large population-based sample of patients with new diagnoses of diabetes, there are some limitations to our data. First, to the extent that the primary care clinical records may have under-captured some comorbidities (particularly likely for conditions like dementia, chronic pain, or depression), we may have underestimated the proportion of individuals with multiple comorbidities and thus overstated the proportion who were otherwise fit. Second, we may have underestimated rates of deintensification as we don’t know if insulin doses had been decreased or if patients were told to split pills after receiving their prescriptions. Third, although some might see it as a weakness that we used prescription records rather than pharmacy dispensation records, we would argue that this is a strength since it permits us to capture physician intent without the data being confounded by primary non-adherence (patients not filling their prescription). Fourth, while there are various scales for evaluating patient frailty, cumulative deficit models (like the eFI we used) have been shown to better predict functional and mortality outcomes than some of the other scales more commonly quoted in the literature [40]. Fifth, although some antihypertensive therapies such as ACE inhibitors or angiotensin receptor blockers are recommended in diabetic individuals with left ventricular dysfunction or significant proteinuria regardless of their SBP, only a very small minority of our cohort had these conditions. Finally, we did not have data on other factors that could influence clinical decision making such as socioeconomic status, specialist involvement in patient care, or patient preferences. Without the fuller picture this data would provide, we elected not to perform multivariate analyses to try to define whether particular comorbidities or patient factors were more likely to be associated with out-of-range HbA1C or SBP levels than other factors to avoid potentially spurious findings.

## Conclusion

In conclusion, although there were statistically significant (but clinically minor) differences in glycemic and hypertensive control across health states, medication changes were uncommon regardless of their health status. Nearly one-third of all diabetic individuals in this cohort had treated HbA1C < 6.5% or SBP < 130 mmHg with little difference in proportions when re-measured 6 months later. On the other hand, nearly half of this cohort exhibited poor control of glucose (HbA1C > 7.5%) or SBP (> 140 mmHg), with again little change in those proportions 6 months later despite frequent primary care visits. Our study thus highlights the need for personalizing treatment targets in adults with type 2 diabetes and implementing diabetes quality improvement strategies [41] that are flexible enough to focus on both the potential over-treatment of multimorbid patients as well as potential under-treatment of healthier patients and to consider both blood pressure as well as glycemic targets. To that end, it is worth noting that a recent Monte Carlo-based Markov Model decision analysis confirmed that care to achieve individualized treatment targets was cost-saving and associated with a small improvement in quality of life [42].

## Additional file

**Additional file 1.** Glycemic and blood pressure levels in the first year after diagnosis of diabetes in those patients with active prescriptions at the time of index measurements.
