# Supplementary material for: Control of glycemia and blood pressure in British adults with diabetes mellitus and subsequent therapy choices: a comparison across health states
Source: Cardiovasc Diabetol. 2018 Feb 12;17:27. doi: 10.1186/s12933-018-0673-4 (PMC5808447; doi:10.1186/s12933-018-0673-4)
Supplement: Supplementary file 1 — Additional file 1. Glycemic and blood pressure levels in the first year after diagnosis of diabetes in those patients with active prescriptions at the time of index measurements. [file 12933_2018_673_MOESM1_ESM.docx]

**Appendix Table S1: Glycemic and blood pressure levels in the first year after diagnosis of diabetes in those patients with active prescriptions at the time of index measurements**

| **HbA1C:** | **Overall (n=160,322)** | **Otherwise Fit (n=147,702)** | **Mild frailty (n=12,259)** | **Moderate or severe frailty (n=361)** | **P-value** |
| --- | --- | --- | --- | --- | --- |
| <6% | 12.3 (19767) | 12.2 (17959) | 14.2 (1745) | 17.5 (63) | <0.0001 |
| 6.0-6.4% | 14.2 (22809) | 14.1 (20892) | 15.2 (1862) | 15.2 (55) | 0.005 |
| 6.5-6.9% | 17.9 (28659) | 17.8 (26322) | 18.5 (2262) | 20.8 (75) | 0.08 |
| 7.0-7.5% | 14.7 (23625) | 14.8 (21810) | 14.4 (1765) | 13.9 (50) | 0.48 |
| >7.5% | 40.8 (65462) | 41.1 (60719) | 37.7 (4625) | 32.7 (118) | <0.0001 |
| **Systolic Blood Pressure:** |  |  |  |  |  |
| <120 mm Hg | 11 (17663) | 10.8 (15995) | 13.2 (1613) | 15.2 (55) | <0.0001 |
| 120-129 mm Hg | 16.6 (26655) | 16.8 (24806) | 14.6 (1785) | 17.7 (64) | <0.0001 |
| 130-139 mm Hg | 23.5 (37723) | 23.7 (35062) | 21.2 (2604) | 15.8 (57) | <0.0001 |
| 140 mm Hg or greater | 48.8 (78281) | 48.6 (71839) | 51 (6257) | 51.2 (185) | <0.0001 |
